# Influence of Curing on the Strength Development of Calcium-Containing Geopolymer Mortar

**DOI:** 10.3390/ma6115069

**Published:** 2013-11-07

**Authors:** Xueying Li, Zheng Wang, Zhenzhen Jiao

**Affiliations:** School of Civil Engineering, Harbin Institute of Technology, Harbin 150006, China; E-Mail: wangz58@163.com

**Keywords:** geopolymer mortar, fly ash, calcium, curing conditions, strength development

## Abstract

This paper investigated the curing effects on the mechanical properties of calcium-containing geopolymer mortar. Three precursors are used: Class C fly ash, Class F fly ash plus calcium hydroxide and Class F fly ash plus slag. Curing conditions included: (1) standard curing at 20 ± 3 °C and RH 95% (C); (2) steam curing at 60 °C for 24 h (S); (3) steam curing at 60 °C for 6 h (S6); and (4) oven curing at 60 °C for 24 h (O), then the latter three followed by the standard curing. Under the standard conditions, the flexural strength and compressive strength of Class C fly ash geopolymer mortars developed quickly until the age of 7 days, followed by a gradual increase. Specimens with Class F fly ash plus Ca(OH)_2_ showed slow increase till the age of 28 days. Under these non-standard conditions (2–4), all specimens showed higher 3-day strength, while later strengths were either higher or lower than those in standard conditions, depending on the type of the precursor.

## 1. Introduction

Geopolymers are being promoted as sustainable construction materials [1,2]. Their mechanical properties are influenced by factors including raw materials, the activator type, water-to-ash ratio and curing conditions. They can be made of metakaolin, fly ash and slag [3,4]. However, metakaolin is expensive and is not used widely in the construction industry. Alkali-activated slag and the alkali-activated fly ash have attracted great research interest [5,6,7,8]. The products of alkali-activated fly ash are calcium silicate hydrate (C–S–H) [9,10,11,12] and amorphous hydrated alkali-aluminosilicate [13,14,15,16,17]. Efforts have been made to investigate geopolymers based on fly ash [18,19].

Fly ash is a by-product of coal burning for the electricity generation. It can be categorized into Class F fly ash (low Calcium) and Class C fly ash (high Calcium). A few studies concluded that Class C fly ash has pozzolantic and cementitious properties [20,21,22]. Calcium resulted in the formation of hydrate C–S–H in addition to the geopolymer gel, enhancing the mechanical strength of the hardened matrix [21,22]. In a geopolymer made using Class C fly ash, curing at ambient and elevated temperature could produce higher strength compared to a geopolymer made using Class F fly ash [20,22]. This conclusion contradicts other study results [23,24]. Therefore, great attention has been paid to calcium’s effects.

Another important factor is the curing conditions. To obtain high strength in fly ash geopolymer, curing temperature at 40–75 °C is normally required [25,26,27]. This high temperature can be used to make the building block; however, it is difficult to construct in the field construction practices. A number of researchers, therefore, have tried to study the strength development of fly ash geopolymer under the ambient temperature [17,19,22]. A variety of additives such as ground granulated blast furnace slag, calcium hydroxide, flue gas desulfurization gypsum and Portland cement have been used [26].

This study aimed to investigate the effect of curing conditions on the strength development of calcium-containing geopolymer mortar.

## 2. Experimental

### 2.1. Materials

Fly ash was used as the aluminosilicate source material for geopolymer. Slag or calcium hydroxide was blended with Class F fly ash. Class C fly ash (CFA) used in this study was obtained from Harbin Acheng Suibao Thermoelectric Power Plant (Harbin, China) and Class F fly ash is from the Harbin 3rd Thermoelectric Power Plant (Harbin, China). The chemical composition of fly ash and slag were given in Table 1. The reagents used to prepare the activators were of laboratory grade NaOH pellets (96 wt % purity) and the sodium silicate solution, which had a composition of 10.5 wt % Na_2_O, 30.5 wt % SiO_2_ and 59 wt % H_2_O, were supplied by Julide Chemical Co., Langfang, China.

**Table 1:** Chemical composition of fly ash and slag.

Oxide	Percentage (wt %)		
	Class C fly ash	Class F fly ash	slag
SiO_2_	48.2	62.29	31.23
Al_2_O_3_	18.4	15.94	17.16
Fe_2_O_3_	3.7	6.24	1.88
CaO	19.6	7.92	38.66
MgO	1.1	1.57	8.6
SO_3_	1.7	–	–
f-CaO	5.2	–	–

### 2.2. Design of Mix Proportion

In order to study the effect of different curing conditions of on different material systems geopolymer, three binding material systems were studied: Class C fly ash geopolymer mortar at the mass ratio of water to fly ash 0.35 and 0.40, represented by CF35 and CF40, Class F fly ash mixed with calcium hydroxide geopolymer mortar at the mass ratio of water to fly ash 0.35 (FFC35), Class F fly ash with slag geopolymer mortar at the mass ratio of water to ash (fly ash + slag) 0.35 (FFS35). The mass ratio of NaOH to ash (fly ash + slag) was 0.058. The mass ratio of ash (fly ash + slag) to sand was 0.5. The mix proportions of materials were presented in Table 2.

**Table 2:** Mix proportion of geopolymer mortar.

Code	Mole ratio					Mass ratio	NaOH/Ash
	SiO_2_/Al_2_O_3_	Na_2_O/Al_2_O_3_	Na_2_O/SiO_2_	CaO/SiO_2_	H_2_O/Na_2_O	Ash/Sand	
CF35	5.38	0.71	0.13	0.46	15.14	0.5	0.058
CF40	5.38	0.71	0.13	0.46	17.31	0.5	0.058
FFC35	7.72	0.82	0.11	0.17	15.14	0.5	0.058
FFS35	7.31	0.82	0.11	0.17	15.14	0.5	0.058

### 2.3. Specimen Preparation

To prepare geopolymer specimens, fly ash (Class C fly ash or Class F fly ash and Ca(OH)_2_ or Class F fly ash and slag) and the sand were mixed for 5 min, after which the activating solution was added and mixed for 2.5 min at a slow rate and for another 2.5 min at a fast rate, respectively. The mortar was cast into prismatic molds with a dimension of 40 mm × 40 mm × 160 mm, vibrated for 1 min to remove entrained air and sealed with a film to prevent moisture loss from the surface.

## 3. Results and Discussion

### 3.1. Strength Development of Class C Fly Ash Geopolymer Mortar

**Figure 1:**
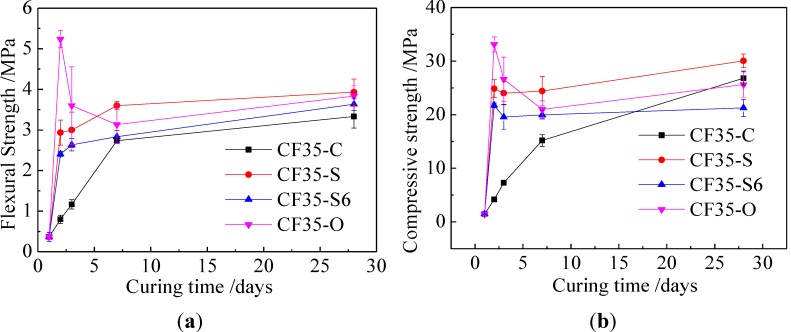
Effect of curing conditions on strength of Class C fly ash geopolymer at W/F0.35. (**a**) Flexural strength; and (**b**) compressive strength.

**Figure 2:**
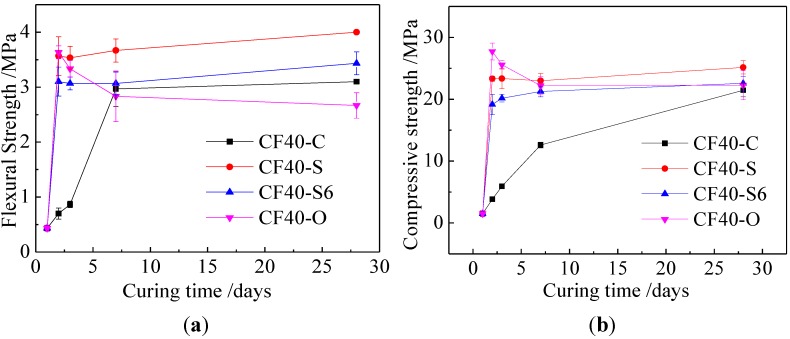
Effect of curing conditions on strength of Class C fly ash geopolymer at W/F0.40. (**a**) Flexural strength; and (**b**) compressive strength.

For Class C fly ash geopolymer mortar with water to ash ratio of 0.35 (CF35-C), Figure 1a,b showed the effect of curing conditions on their flexural and compressive strength, respectively. Before the age of 7 days, the non-standard curing resulted in much higher strength than the standard curing. After steam curing for 24 h and 6 h (CF35-S and CF35-S6), strength increased rapidly at the age of 1 day, then strength developed slowly. The compressive strengths corresponding to steam curing for CF35-S6 and CF35-S were 21.75 MPa and 24.87 MPa, respectively. Those values were 5.2 times and 5.9 times as much as that after standard curing for CF35-C, respectively. The compressive strength of geopolymer mortar cured in the oven (CF35-O) for 2 days increased to the maximum value then shut down later, and compressive strengths were 33.1, 26.62, 20.98 MPa at the ages of 2, 3, 7 days, respectively, which were 7.9, 3.6, 1.4 times correspondingly as much as compressive strength under the standard curing. After 7 days, the strength of non-standard cured specimens was comparable or even lower than those from standard curing, the corresponding compressive strengths (CF35-S and CF35-S6) were 21.27 MPa and 30.05 MPa at the age of 28 days, just 0.8 times and 1.1 times as much as that under standard curing, respectively. With higher water to ash ratio, a similar phenomenon was observed. From Figure 2, for the mass ratio of water to fly ash at 0.40 geopolymer mortar (CF40), the strength development had similar to the geopolymer mortar at W/F 0.35. However, the 28-day strength was lower than that of CF35. In summary, for the Class C fly ash geopolymers investigated in this study, these non-standard curing conditions resulted in higher early age (7 days) strength, while the standard curing was beneficial to longer-term strength development.

The higher early-age strength at high temperature curing (60 °C) was attributed to the increased dissolution rate of SiO_2_ and Al_2_O_3_ from precursors, which increased the rate of geopolymer formation [28,29]. When the geopolymer samples were later cured in standard conditions, insufficient SiO_2_ and Al_2_O_3_ species were released because of the earlier consumption of the activators, thus resulting in slow strength development. Furthermore, sufficient water was available for geopolymer formation during oven curing, while, after this curing, not enough water in the specimens was present for further geopolymerization and thus for strength development.

### 3.2. Strength Development of Class F Fly Ash Geopolymer Mortar

**Figure 3:**
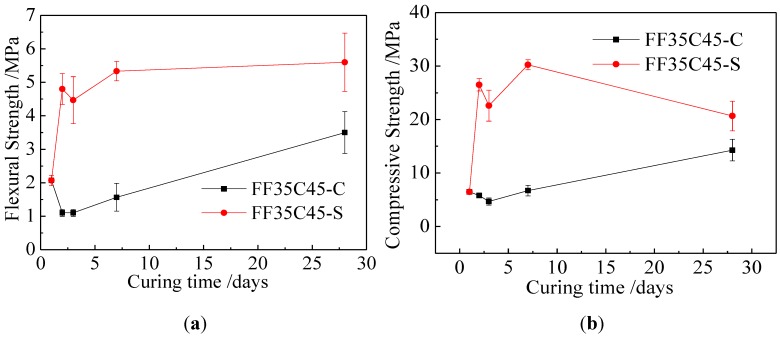
Effect of curing conditions on strength of Class F fly ash mixed with Ca(OH)_2_ geopolymer mortar. (**a**) Flexural strength; and (**b**) compressive strength.

The effects of curing conditions on the strength of Class F fly ash mixed with calcium hydroxide geopolymer mortar were shown in Figure 3. The compressive strength and the flexural strength of geopolymer mortar after being demolded cured at standard temperature for 1 day were up to 6.48 MPa and 2.07 MPa. These high strengths were attributed to the addition of Ca(OH)_2_ which enhanced the concentration of OH^−^ to dissolve the Class F fly ash. The compressive strength decreased in the first 3 days and then increased up to 14.28 MPa at the age of 28 days under standard curing. Under the steam curing conditions, the compressive strength fluctuated during the curing period and compressive strength reached the maximum value of 30.23 MPa at the age of 7 days. Steam curing provided higher strength than standard curing for geopolymer mortars of Class F plus Ca(OH)_2_.

**Figure 4:**
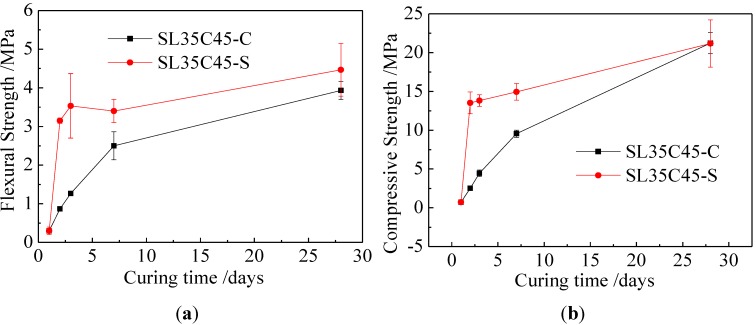
Effect of curing conditions on strength of Class F fly ash-slag geopolymer mortar. (**a**) Flexural strength; and (**b**) compressive strength.

The effect of curing conditions on the strength of Class F fly ash with slag geopolymer mortar was shown in Figure 4. In this Figure 4b, the compressive strength of geopolymer mortar prepared by Class F fly ash and slag increased with the increase of curing time under standard curing and steam curing. Besides, the flexural strength under standard curing conditions showed a similar trend. Under steam curing conditions (seen in Figure 4a), both the 3-day and 28-day flexural strengths were higher than those under standard curing conditions, athough the difference of the 28-day strength was negligible. These results indicated that the steam curing increased the early strength of Class F fly ash-slag geopolymer mortar.

## 4. Conclusions

In this study, the effect of curing conditions on the mechanical properties of fly ash-based geopolymer was investigated by measuring the development of compressive and flexural strength. The experimental results led to the following conclusions.

(1) For the Class C fly ash geopolymers, the early-age (<7 days) strength of oven curing was the highest, followed by that from steam curing conditions at 60 °C for 24 h. Both high temperature curing conditions showed higher strength than standard curing conditions at early age. At later ages, however, standard curing showed comparable or higher mechanical strength.
(2) Addition of slag addition and calcium hydroxide was indicated to improve the strength of Class F fly ash geopolymer at early ages under the standard curing conditions.
